# Smoking cessation and exercise: perspectives from smokers with and without mental health problems

**DOI:** 10.3389/fpubh.2025.1589719

**Published:** 2025-05-16

**Authors:** Stefanie E. Schöttl, Laura Scheibner, Anika Frühauf, Prisca Kopp-Wilfling, Monika Edlinger, Bernhard Holzner, Martin Kopp

**Affiliations:** ^1^Department of Sport Science, University of Innsbruck, Innsbruck, Austria; ^2^Department of Psychiatry, Psychotherapy and Psychosomatics, Psychiatry I, Medical University of Innsbruck, Innsbruck, Austria

**Keywords:** smoking, exercise, smoking cessation, mental health, perspectives, barriers

## Abstract

**Introduction:**

Several smoking cessation methods are available, but the approaches were originally developed for the general population and are often applied to people with mental health problems. To address the needs of smokers with and without mental health problems for an exercise-assisted smoking cessation program, it seems necessary to know more about potentially different perspectives on what prevents them from quitting, how to increase motivation to quit, and how such a program is perceived.

**Methods:**

In this study, an online survey was conducted to assess preferences for an exercise-assisted smoking cessation program, reasons for smoking, barriers, motives, and need of support for quitting smoking. A total of 257 smokers took part in the study, 82 reported mental health problems.

**Results:**

In addition to significant differences in sociodemographic and smoking-related characteristics between smokers with and without mental health problems, regression analyses revealed that factors such as age, BMI, and confidence in quitting were associated with smoking cessation behavior in the total sample. Significant differences were found between the two groups in their reasons for smoking, barriers, motives, and support for quitting. While both groups preferred an exercise-assisted smoking cessation program to last 10–11 weeks with a frequency of 2 to 3 days a week, smokers with mental health problems favored shorter exercise sessions and would choose walking or dancing as helpful exercise.

**Conclusion:**

Due to different addiction-related variables and preferences, a special smoking cessation program combined with exercise for smokers with mental health problems should be developed and tested for effectiveness in clinical trials.

## 1 Introduction

Tobacco use, particularly cigarette smoking, is one of the leading and most preventable causes of premature death worldwide (1–3). Cigarette smoking is a well-established risk factor for cardiovascular and respiratory diseases, as well as for several types of cancer (4–6). While smoking rates in the general population have declined over time (3), large-scale epidemiologic surveys (7, 8) have reported that the number of smokers with mental health problems (MHP) remains high. Individuals with MHP are two to four times more likely to smoke than the general population, depending on the type and number of psychiatric diagnoses (9, 10). They also tend to smoke more heavily and have a greater nicotine dependence (9, 11). Several mechanisms may explain the association between MHP and tobacco dependence. Research suggests that both psychiatric disorders and nicotine dependence share common genetic factors, particularly those affecting the brain’s dopamine and nicotine systems (12). People with depression may use smoking to regulate their mood, as nicotine has short-term effects on dopamine release (13, 14). In schizophrenia and other psychotic disorders, smoking is discussed as a way of coping with cognitive deficits and reducing the side effects of antipsychotic medication (15). Moreover, smoking may be used as a weight-control strategy to suppress appetite, which may be particularly relevant for people with eating disorders (16). In addition to increased nicotine dependence, people with MHP experience more withdrawal symptoms after quitting smoking, and have lower quit rates but higher relapse rates than the general population (9, 11). Rasmussen et al. (17) examined self-reported abstinence among smokers with and without severe MHP at a six-month follow-up after participating in a smoking cessation intervention. A significantly lower abstinence rate was found among smokers with severe MHP compared to participants without MHP (17). Although people with MHP are motivated to quit smoking in a similar way to the general population, their confidence in being able to quit is lower (18, 19). Therefore, smoking cessation is a more challenging issue for people with MHP (20).

There are several effective methods to support smoking cessation in people with and without MHP, including psychosocial support, behavioral counseling, nicotine replacement therapies, and medications (e.g., bupropion or varenicline) (2, 10, 21). In addition, newer options such as smartphone apps and e-cigarettes have been explored. E-cigarettes deliver nicotine through vaper rather than burning tobacco, reducing exposure to harmful chemicals in cigarette smoke (22). Some studies suggest that they may help people quit smoking by imitating the behavioral and sensory aspects of smoking while providing controlled nicotine delivery (22). However, the evidence remains mixed as e-cigarettes may prolong nicotine dependence and there are concerns about potential long-term health risks (23). Exercise is also used as an add-on therapy in smoking cessation and has several benefits for people with and without MHP (24, 25): It can help to (a) alleviate withdrawal symptoms and cigarette cravings (26), (b) reduce the adverse effects of smoking on the cardiovascular and respiratory systems (27), (c) improve mood, well-being and quality of life (26, 28), (d) reduce stress and symptoms of depression and anxiety (29), (e) decrease smoking cessation-related weight gain (30), (f) distract attention from cigarette cravings (25), (g) improve self-esteem and confidence in general, thereby facilitating the smoking cessation process (31), and it can further be a useful way to pass time during boredom (19), as well as being a cost-effective treatment that is easy to access (32).

A closer look at the studies examining the effects of exercise on cigarette cravings and withdrawal symptoms in temporarily abstinent smokers or investigating exercise as add-on therapy in smoking cessation revealed two issues: First, the exercise sessions used in the studies varied in duration, type and intensity (25, 33), and the strength of the effects may depend on the characteristics of the exercise (26, 34). Secondly, the literature has focused more on studies investigating exercise as an adjunct to smoking cessation in people without MHP, and fewer studies have addressed the topic in people with MHP (24, 26). Efforts to support people to quit smoking are primarily aimed at the general public, as people with MHP are a hard-to-reach group for health interventions and differ in some aspects from the general population (35, 36). Low socioeconomic status, limited education and low social support among people with MHP may be associated with inadequate access to preventive medicine, physical health screening as well as medical care (37). There are also common misconceptions about smoking and quitting smoking in people with MHP: In psychiatric settings, where patients tend to smoke due to boredom, distraction or peer pressure, smoking is often tolerated by staff and facilitated by organized smoking breaks (9, 11, 36). In addition, healthcare professionals fear that additional smoking cessation may affect the treatment of the patient’s MHP (38). It is also believed that cigarettes may help as self-medication and that smoking cessation should only be implemented at a stable stage, when the patient’s mental health has improved (9, 38). In addition, the various smoking cessation approaches originally developed for the general population are often applied to people with MHP without being prior evaluated in this target group (39).

To better address the needs of smokers with MHP and to understand how and where they differ from smokers without MHP, it is necessary to explore what prevents them from quitting, what might increase their motivation to quit, and how they perceive a smoking cessation program combined with exercise. The current literature has examined individual aspects of smoking behavior, such as reasons for smoking (40), motivation to quit (41, 42), and barriers to quitting (42, 43) -primarily in the general population. Similarly, some studies have focused on these aspects in people with MHP, examining, for example, reasons for smoking (19, 44), motivation to quit (18, 19, 45), and barriers to quitting (44, 46). However, most of these studies have addressed only isolated aspects, either in populations with or without MHP. Only a few studies, such as Baker et al. (37) and Twyman et al. (20), have attempted to compare single or multiple dimensions between smokers with and without MHP, but even these are limited to a subset of factors (e.g., reasons for smoking, barriers to quitting) rather than providing a comprehensive overview. This gap underscores the need for a holistic examination of reasons for smoking, barriers, motivation and support for quitting in order to develop tailored, evidence-based interventions that address the unique challenges of smokers with mental health conditions. Furthermore, there is also a gap in the literature regarding exercise-assisted smoking cessation programs. To the best of our knowledge, no study has comprehensively investigated and compared preferences for a smoking cessation program combined with exercise among individuals with and without MHP. Often, smoking cessation approaches developed for the general population are applied to people with MHP without prior evaluation in this population (39). Understanding these preferences is essential before planning further intervention studies: the success of smoking cessation may depend on tailoring the smoking cessation program and the exercise component to the needs and preferences of individuals, particularly those with MHP. Therefore, our study has two aims: First, to comprehensively investigate the reasons for smoking, barriers, motivation and support to quit smoking, and second, to identify preferences for a smoking cessation program combined with exercise in smokers with self-reported MHP compared to smokers without MHP.

## 2 Materials and methods

### 2.1 Study design and procedure

An online survey was developed for data collection. A pretest was then conducted to assess its clarity, readability, and overall comprehensibility, with the aim of optimizing participants’ understanding and improving response rates (47). The online survey was addressed to individuals who smoked daily for at least 1 year and were 18 years or older. People with self-reported mental health problems were also invited to participate, as this was clearly stated in the introduction and invitation text of the questionnaire. The online survey was promoted through a variety of targeted channels within the German-speaking community to engage individuals specifically interested in smoking cessation, thereby increasing both sample representativeness and data quality. Specifically, the survey was posted on online forums with a clear focus on health, mental and smoking cessation, such as dedicated smoking cessation forums, depression self-help forums, and anxiety/panic self-help forums. In addition, the survey link was shared on the social media platform Facebook, where it was posted in several groups dedicated to topics such as mental illness, mental health, and smoking cessation. The survey was also distributed via mass email to all students at the University of Innsbruck, targeting a diverse student population and ensuring broad reach. At the beginning of the questionnaire, participants were informed about the inclusion criteria, the aim and duration of the survey (approximate duration of 10 min), privacy, and data protection. Afterwards, they had to confirm (by ticking a box) that they had read and understood the information and agreed to participate in the survey. The questionnaire was only available in German. No incentives were offered for participation. The survey was conducted according to the “ethical guidelines for surveys” approved by the Institutional Review Board (IRB) of the Department of Sport Science as well as the Board for Ethical Issues (BfEI) of the University of Innsbruck in accordance with the Declaration of Helsinki (#17/2025, date: 20.02.2025). To comply with the ethical guidelines, there were no mandatory fields in the questionnaire.

### 2.2 Measures

The survey consisted of four sections: (1) Sociodemographic data, health and physical activity (PA) behavior, (2) smoking-related characteristics of participants, (3) reasons for smoking, barriers, motives and support to quit smoking, (4) preferences for a smoking cessation program and preferences for exercise in combination with smoking cessation.

#### 2.2.1 Sociodemographic data, health and PA assessment

At the beginning of the questionnaire, sociodemographic characteristics were queried, and participants were asked whether they had been diagnosed with a mental health disorder. If they agreed, they could specify the condition. Two items from the brief version of the World Health Organization quality of life questionnaire (WHOQOL-BREF) (48) were used, to assess quality of life and general health. On a 5-point Likert scale, participants indicated how satisfied they were with their health (from very dissatisfied to very satisfied) and how good they rated their quality of life (from very poor to very good) (49). To collect the subjective self-reported PA level, the “short last 7 days self-administered format” of the International Physical Activity Questionnaire (IPAQ-SF) was used (50). Following the IPAQ-calculation guidelines (51), PA in MET-minutes per week and PA level in categories (low, moderate, high) were automatically calculated. The validity and reliability of the IPAQ have been provided previously (52).

#### 2.2.2 Smoking-related characteristics

The Fagerström Test for Cigarette Dependence (FTCD) was used to assess cigarette dependence (53). The questionnaire consists of six items, from which a total score (from 0 to 10) was calculated. The total score was categorized to indicate the degree of cigarette dependence (very low, low, medium, high, very high) (53). The validity and reliability of the questionnaire were tested (54). Smoking-related characteristics such as years of smoking, cigarettes per day, quit attempts, and confidence in becoming smoke-free (10-point Likert scale from 0 to 100%) were also asked. To determine the readiness to quit smoking and to classify participants into the Stages of Change (55), participants were asked if they intended to quit smoking. Based on their responses, participants were assigned to the stage of pre-contemplation (“no”), contemplation (yes, thinking about quitting smoking within the next 6 months”) or preparation (“yes, thinking about quitting smoking within the next 30 days”) (55, 56). In addition, study participants were asked if a physician had ever recommended that they participate in a smoking cessation program.

#### 2.2.3 Reasons for and barriers, motives and support to quit smoking

Individuals could select several reasons for their smoking behavior and were also asked about their barriers to smoking cessation. Furthermore, participants could choose different reasons that would motivate them to quit smoking, and they could also choose items that would help them to quit smoking (multiple answers were allowed).

#### 2.2.4 Preferences for a smoking cessation program in combination with exercise

Participants indicated the duration (weeks), frequency (days/week) and setting (alone or in a group) they would prefer for a smoking cessation program. Study participants were also asked if they would participate in a smoking cessation program that included exercise sessions. They were able to provide input on the type (multiple answers were possible), location (indoor, outdoor), frequency (days per week) and duration (minutes) of exercise.

### 2.3 Statistical analyses

Descriptive data are presented as means (M) and standard deviations (SD) for continuous variables and as percentages for categorial (nominal or ordinal) variables. To analyze differences in sociodemographic and smoking-related characteristics between smokers with and without mental health problems, statistical tests were selected based on the measurement scale of the variables. For categorical variables, χ^2^ tests were used to assess group differences. For continuous variables, independent t-tests were used when normality and homogeneity of variances were assumed. Homogeneity of variances was tested using Levene’s test (*p* < 0.05). If the assumption of equal variances was violated, the Welch test was used, which provides a more robust alternative to the standard t-test under heteroscedasticity. Nonparametric tests, such as the Mann–Whitney U test, were not required because the assumptions for parametric tests were met for the continuous variables analyzed.

Differences in sociodemographic and smoking-related characteristics may affect group comparisons. To control for potential confounding variables, regression analyses were performed. Two binary logistic regression models were performed to identify predictors of “readiness to quit smoking” (yes/no) and “health complaints due to smoking” (yes/no). For the dependent variable “readiness to quit smoking,” the data from the stages of change were transformed into “no” (precontemplation) and “yes” (contemplation and preparation). In addition, factors associated with nicotine dependence (measured by the FTCD) and confidence in quitting smoking were examined using two multiple linear regression models. Independent variables included sociodemographic and health-related characteristics (e.g., mental health problems, gender, education, age, BMI, assessment of quality of life and general health) as well as smoking-related characteristics (e.g., cigarette dependence, number of quit attempts, confidence in quitting smoking, and health complaints due to smoking). To validate the regression models and ensure the robustness of the results, diagnostic procedures were performed, including the identification of outliers (Leverage, Cook’s Distance), assessment of multicollinearity, and verification of model assumptions (57). For the linear regression models, we also checked the normality of the residuals and tested their independence using the Durbin-Watson statistics.

To analyze the reasons for smoking, the barriers, the motivation and the support to quit smoking, the factors in each of these areas were grouped into distinct categories. The categories were defined based on theoretical frameworks, theories and relevant literature. A detailed explanation of the derivation of the categories is provided in the Supplementary Tables 1–4. The defined categories allowed a structured comparison between smokers with and without MHP. The frequencies (percentages) of reported factors within each category were used to calculate the mean percentages for each category to identify significant differences between groups. Group differences were assessed using χ^2^-tests.

Data were analysed using IBM SPSS Statistics (version 28) and statistical significance was reached if *p*-values < 0.05.

## 3 Results

### 3.1 Study sample

After data cleaning, a total of 257 smokers were included in the study analysis. 175 of the respondents reported no mental health problems (group S: smokers), while 82 people mentioned mental health problems (group SMHP: smokers with mental health problems). The following mental health problems were reported by participants: attention deficit/hyperactivity disorder, anxiety disorder, agoraphobia, panic disorder, obsessive-compulsive disorder, borderline disorder, bipolar disorder, depression, depressive episode, dissociative disorder, personality disorder, post-traumatic stress disorder, eating disorder, bulimia nervosa, schizophrenia, paranoid schizophrenia, schizoaffective disorder. All self-reported mental health problems could also occur as comorbidities.

### 3.2 Sociodemographic data, health and PA

Respondents with MHP had a significantly higher age, higher BMI and lower education than smokers without MHP (Table 1). No significant differences could be seen in marital status and PA level. Smokers without MHP (group S) had a significantly better assessment of their quality of life and their health than smokers with MHP (group SMHP).

**Table 1:** Sociodemographic characteristics, health and PA assessment of smokers without (group S) and with (group SMHP) mental health problems.

Variable % or mean ± SD	Group S (*n* = 175)	Group SMHP (*n* = 82)	Total (*n* = 257)	*p*-value
Gender (%)				**0.015** ^**a**^
Female	62.9%	78%	67.7%	
Male	37.1%	22.0%	32.3%	
Age (mean ± SD)	29.3 ± 11.2	37.2 ± 13.1	31.8 ± 12.4	**<0.001** ^**c**^
BMI (mean ± SD)	23.5 ± 4.1	28.4 ± 8.5	25.1 ± 6.3	**<0.001** ^**c**^
Marital status (%)				0.485^a^
Single	51.4%	56.1%	52.9%	
Partner/married	48.6%	43.9%	47.1%	
Education (%)				**<0.001** ^**a**^
School-leaving qualification	6.9%	34.1%	15.6%	
High School	52.6%	23.2%	43.2%	
Vocational training	5.7%	28%	12.8%	
University degree	34.9%	14.6%	28.4%	
PA level (IPAQ-SF)				0.120^a^
Low	14.3%	20.6%	16.2%	
Moderate	18.4%	27.0%	21.0%	
High	67.3%	52.4%	62.9%	
Assessment quality of life*	3.9 ± 0.9	2.9 ± 0.9	3.6 ± 1.0	**<0.001** ^**b**^
Assessment health*	3.8 ± 0.9	2.7 ± 1.0	3.4 ± 1.1	**<0.001** ^**c**^

Significant differences are shown in bold. * = participant number varied; IPAQ-SF, International Physical Activity Questionnaire-Short Form.

a Chi^2^-test.

b Independent *t*-test.

c Welch-test.

### 3.3 Smoking-related characteristics

Participants from group SMHP reported a significantly higher nicotine dependence, more years of regular smoking, more cigarettes per day and more health complaints due to smoking compared to group S. They further reported a non-significant higher number of quit attempts but less confidence in becoming smoke-free (Table 2). The variables of the age they started smoking, the stages of change, and if people attempted to quit smoking did not significantly differ between both groups. Only 4.1% of group S and 12.8% of group SMHP had been advised by a physician to participate in a smoking cessation program. A significant difference between groups could be observed.

**Table 2:** Smoking related characteristics of smokers without (group S) and with (group SMHP) mental health problems.

Variable % or mean ± SD	Group S (*n* = 175)	Group SMHP (*n* = 82)	Total (*n* = 257)	*p*-value
Cigarette dependence (FTCD)	2.8 ± 2.4	5.2 ± 2.6	3.5 ± 2.7	**<0.001** ^**b**^
Age of start smoking	16.8 ± 3.2	16.4 ± 3.9	16.7 ± 3.4	0.346^b^
Years of regular smoking	11.1 ± 10.4	19.5 ± 13.7	13.8 ± 12.2	**<0.001** ^**c**^
Cigarettes per day	11.5 ± 7.9	17.1 ± 8.9	13.3 ± 8.6	**<0.001** ^**b**^
Health complaints due to smoking (Yes)	26.3%	61%	37.4%	**<0.001** ^**a**^
Recommendation of physician to participate in smoking cessation (Yes)	4.1%	12.8%	6.9%	**0.012** ^**a**^
Stages of change*				0.661^a^
Precontemplation	54.4%	49.4%	52.8%	
Contemplation	33.3%	39.2%	35.2%	
Preparation	12.3%	11.4%	12.0%	
Quit attempt (Yes)	70.4%	80.8%	73.7%	0.086^a^
Number of quit attempts	2.3 ± 3.4	3.6 ± 6.3	2.7 ± 4.5	0.071^c^
Confidence in quitting smoking	5.6 ± 3.0	4.3 ± 2.8	5.2 ± 3.0	**0.002** ^**b**^

Significant differences are shown in bold; * = participant number varied; FTCD, Fagerström Test for Cigarette Dependence.

a Chi^2^-test.

b Independent *t*-test.

c Welch-test.

### 3.4 Predictors of smoking behavior: controlling for confounding variables

Two binary logistic regressions were performed to identify predictors of (1) “readiness to quit smoking” (yes/no) and (2) “health complaints due to smoking” (yes/no). The model for “readiness to quit smoking” was statistically significant, χ^2^(12) = 80.268, *p* < 0.001, with a good model fit (χ^2^(8) = 4.558, *p* = 0.804) (Hosmer-Lemeshow test). Nagelkerke’s R^2^ of 0.432 indicates a moderate effect size, reflecting a substantial amount of variance explained by the model. Eleven variables were included in the regression model, with four variables significantly predicting readiness to quit smoking. Smokers were more likely to be ready to quit if they were female, reported more quit attempts, and were more confident about quitting. Conversely, a higher BMI was associated with a lower readiness to quit smoking (see Table 3).

**Table 3:** Results of the binary logistic regression model investigating associations between readiness to quit smoking and smoking-related health complaints in the total sample and several variables (*n* = 205).

Variable and categories	Readiness to quit smoking			Smoking-related health complaints		
	Regression coefficient B	Odds ratio Exp (B)	Sig.	Regression coefficient B	Odds ratio exp (B)	Sig.
Mental health problem						
Yes	−0.149	0.861	0.732	0.691	1.995	0.104
No (Reference)						
Gender						
Female	0.978	2.660	**0.017**	0.151	1.163	0.703
Male (Reference)						
Smoking-related health complaints						
Yes	0.367	1.444	0.365	/	/	/
No (Reference)						
Education						
Low (Reference)						
Middle	−0.527	0.590	0.392	0.596	1.815	0.298
High	0.118	1.125	0.861	0.292	1.339	0.649
Age	−0.034	0.966	0.082	0.000	1.000	0.992
BMI	−0.077	0.926	**0.048**	0.048	1.049	0.194
Assessment of quality of life	−0.443	0.642	0.074	0.101	1.107	0.692
Assessment of health	−0.203	0.816	0.398	−0.903	0.406	**<0.001**
Cigarette dependence (FTCD)	0.122	1.130	0.149	0.155	1.168	0.058
Number of quit attempts	0.521	1.684	**<0.001**	0.054	1.056	0.277
Confidence in quitting smoking	0.348	1.417	**<0.001**	0.183	1.201	**0.013**

The binary logistic regression model for “health complaints due to smoking” was again statistically significant χ^2^(11) = 67.636, *p* < 0.001. Goodness-of-fit was assessed using the Hosmer-Lemeshow test, which indicated an acceptable fit (χ^2^(8) = 15.166, *p* = 0.056). The Nagelkerke’s R^2^ value of 0.383 indicates a small effect size. Ten variables were included in the regression analysis, of which two were significantly associated with smoking-related health complaints. Smokers who rated their general health more positively reported fewer smoking-related health complaints. Furthermore, smokers who were more confident about becoming smoke-free tended to report more health complaints.

The multiple linear regression model for “cigarette dependence” was statistically significant, *F*(11, 193) = 9.9, *p* < 0.001. The model explained a substantial amount of variance, with an R^2^ of 0.361 (adjusted R^2^ = 0.324), indicating good fit. Ten variables were entered into the model, with two variables showing significant effects. Older age was associated with higher levels of cigarette dependence. In addition, higher confidence in quitting smoking was associated with lower cigarette dependence (see Table 4).

**Table 4:** Results of the multiple linear regression model investigating associations between cigarette dependence and confidence in quitting smoking of total sample and several variables (*n* = 205).

Variable and categories	Cigarette dependence			Confidence in quitting smoking		
	Unstandardized b	Standardized β	Sig.	Unstandardized b	Standardized β	Sig.
Mental health problem						
Yes	0.573	0.102	0.154	−0.214	−0.035	0.667
No (Reference)						
Gender						
Female	−0.322	−0.058	0.334	−0.174	−0.029	0.671
Male (Reference)						
Smoking-related health complaints						
Yes	0.693	0.130	0.057	1.104	0.188	**0.014**
No (Reference)						
Education						
Low (Reference)						
Middle	−0.541	−0.104	0.285	−1.058	−0.185	0.089
High	−0.653	−0.116	0.245	−1.492	−0.240	**0.030**
Age	0.054	0.249	**<0.001**	0.001	0.004	0.957
BMI	0.018	0.043	0.541	−0.021	−0.045	0.564
Assessment of quality of life	−0.162	−0.062	0.447	0.537	0.187	**0.039**
Assessment of health	−0.226	−0.094	0.276	0.277	0.106	0.277
Cigarette dependence (FTCD)	/	/	/	−0.342	−0.311	**<0.001**
Number of quit attempts	−0.038	−0.068	0.258	−0.002	−0.004	0.958
Confidence in quitting smoking	−0.226	−0.248	**<0.001**	/	/	/

The multiple linear regression model for “confidence in quitting smoking” was also statistically significant, F(11, 193) = 4.354, *p* < 0.001. The model explained a moderate amount of variance, with an R^2^ of 0.199 (adjusted R^2^ = 0.153), indicating moderate goodness of fit. Ten variables were included in the model and four were found to be statistically significant. Smokers with smoking-related health complaints and a higher quality of life were more confident about quitting. Higher education and cigarette dependence were associated with lower confidence in quitting.

### 3.5 Reasons for smoking, barriers, motivation and support to quit smoking

**Figure 1:**
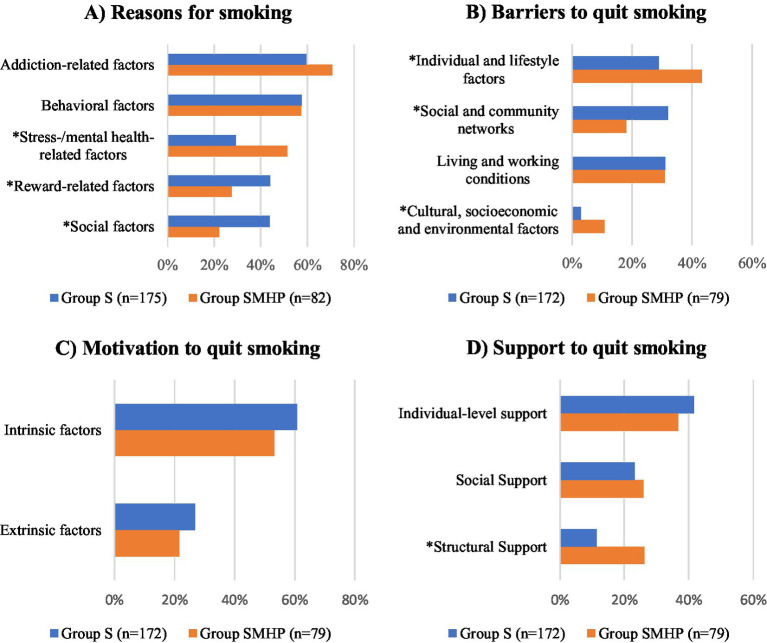
Reasons for smoking **(A)**, barriers **(B)**, motivation **(C)** and support **(D)** to quit smoking for the respective categories shown as mean of reasons (multiple answers possible) for smokers without (group S) and with (group SMHP) mental health problems (Note: significant differences in the categories between the two groups measured by Chi^2^-test are marked with *).

A significantly higher proportion of individuals in group S reported smoking due to social factors (sociability, smoking in a social environment) (*p* < 0.001) and reward-related factors (pleasure, stimulation) (*p* < 0.001) (Figure 1A). In contrast, individuals in the group SMHP were significantly more likely to smoke due to stress- and mental health-related factors (stress, coping with negative mental health symptoms) (*p* < 0.001). No significant differences were found between the two groups regarding addiction-related factors (*p* = 0.080) or behavioral factors (habit, boredom) (*p* = 0.688).

Significantly more people in the SMHP group reported barriers related to individual and lifestyle factors (habit, strong cravings, fear of changes in personality and mood changes, fear of weight gain, sadness, low self-confidence, exhaustion) (*p* < 0.001) and cultural, socioeconomic and environmental factors (lack of appropriate local services, lack of knowledge about smoking cessation programs) (*p* = 0.003). Significantly more participants in group S identified social and community network factors (social environment smokes, parties, peer pressure, no support person) (*p* < 0.001) as barriers to smoking cessation. There were no significant differences between the groups for living and working conditions (boredom, stress, lack of time) (*p* = 0.755) (Figure 1B).

In both groups, intrinsic factors, such as concern for one’s health and the desire to improve physical fitness, were more frequently reported as motives for quitting smoking than extrinsic factors, including financial incentives, family influence, relationship or partner-related reasons, and pregnancy. However, no significant differences in the distribution of intrinsic (*p* = 0.253) or extrinsic (*p* = 0.104) motives were observed between smokers with and without mental health problems (Figure 1C).

Furthermore, no significant differences were found between the two groups regarding support for quitting smoking through individual-level support (distractions, other activities, financial incentives) (*p* = 0.449) and social support (family, friends, physician) (*p* = 0.180). However, structural support factors (*p* < 0.001) such as replacement products (chewing gum, e-cigarettes), access to cessation programs, and the availability of various cessation support options were reported significantly more often by individuals in the SMHP group compared to those in the S group (Figure 1D).

Supplementary Tables 1–4 in the supplementary material show the factors for each category of reasons for smoking, barriers, motivation, and support to quit smoking.

### 3.6 Preferences for a smoking cessation program in combination with exercise

Both groups mentioned that the smoking cessation program should last 10 to 11 weeks and should be held on two to 3 days per week, without significant difference between groups. More participants from group S would be willing to complete a smoking cessation program alone while a higher number of respondents from group SMHP would prefer to complete a program in a group setting.

Over 70% of both groups would be willing to participate in a smoking cessation program combined with exercise (Table 5). Both groups would prefer the exercise as part of the smoking cessation program on 2 to 3 days a week. Group S respondents preferred longer exercise sessions (e.g., 60 min) compared to group SMHP (e.g., 48 min). About half of the participants from both groups indicated that the exercise could take place outdoors and indoors. Individuals with MHP most frequently selected walking, dancing, workouts, swimming and yoga as their preferred sports. Smokers without MHP chose yoga, running, workouts, ball sports and swimming. Significantly more smokers from group SMHP compared to group S would choose dancing and (Nordic) walking as their preferred sports. A significantly higher number of participants from group S selected running compared to group SMHP.

**Table 5:** Preferences for a smoking cessation program and a smoking cessation program in combination with exercise by smokers without (group S) and with (group SMHP) mental health problems.

Variable % or mean ± SD	Group S (*n* = 159)	Group SMHP (*n* = 73)	Total (*n* = 232)	*p*-value
Smoking cessation program				
Alone	61.6%	52.1%	58.6%	0.169^a^
In a group	38.4%	50.7%	42.2%	0.078^a^
Duration (weeks) of program*	10.2 ± 5.3	11.4 ± 8.6	10.6 ± 6.5	0.220^b^
Frequency (days per week) of program*	2.5 ± 1.7	2.8 ± 1.5	2.6 ± 1.6	0.232^b^
Participating in smoking cessation program in combination with exercise (Yes)*	72.2%	76.8%	73.6%	0.470^a^
Frequency of exercise (days per week)*	2.5 ± 1.6	2.7 ± 1.9	2.6 ± 1.7	0.252^b^
Duration (minutes) of exercise*	60.3 + 35.7	48.0 + 33.7	56.2 + 35.5	**0.021** ^**b**^
Place of exercise*				0.079^a^
Outdoor	48.3%	33.3%	43.5%	
Indoor	4.2%	9.1%	5.7%	
Both (outdoor or indoor)	47.6%	57.6%	50.7%	
Type of sport				
(Nordic) Walking	32.7%	46.6%	37.1%	**0.042** ^**a**^
Running	42.8%	23.3%	36.6%	**0.004** ^**a**^
Aerobic	16.4%	23.3%	18.5%	0.207^a^
Workout (fitness, muscle strength)	40.9%	43.8%	41.8%	0.672^a^
Ball sports (e.g., soccer, basketball)	36.5%	24.7%	32.8%	0.075^a^
Spinning	14.5%	13.7%	14.2%	0.877^a^
Yoga	45.9%	41.1%	44.4%	0.493^a^
Pilates	23.3%	20.5%	22.4%	0.644^a^
Aqua gymnastics	13.8%	21.9%	16.4%	0.122^a^
Swimming	34.6%	41.1%	36.6%	0.340^a^
Climbing	27.0%	20.5%	25.0%	0.289^a^
Dancing	30.2%	43.8%	34.5%	**0.042** ^a^

Significant differences are shown in bold. = participant number varied.

a Chi^2^-test.

b Independent *t*-test.

## 4 Discussion

The aim of our study was to identify reasons for smoking, barriers, motives and support for quitting smoking and to investigate preferences for a smoking cessation program combined with exercise in smokers with and without self-reported MHP. Significant differences in sociodemographic and smoking-related characteristics were observed between the two groups. Regression analyses showed that factors such as age, BMI, and confidence in quitting were associated with smoking cessation behavior in the total sample. In addition, smokers with and without MHP differed in their reasons for smoking, perceived barriers, motives and support for quitting. Over 70% of respondents in both groups expressed a willingness to participate in an exercise-assisted smoking cessation program. While both groups preferred a program lasting 10 or 11 weeks with sessions held 2 to 3 times per week, smokers with MHP preferred shorter exercise sessions and lower intensity exercise sessions than smokers without MHP.

Differences in sociodemographic, health- and smoking-related characteristics between participants with and without MHP in our study are consistent with previous research. As in other studies, participants with MHP had lower education levels (35), higher BMI (58), lower perceived quality of life and health (59) and higher nicotine dependence (9). People with MHP often have higher nicotine dependence as they may use smoking to cope with stress and relieve symptoms of mental health disorders (9, 60). This increased dependence and cigarette consumption may contribute to more smoking-related health problems, as there is strong evidence linking nicotine dependence to poorer physical and mental health (61, 62). Our findings also confirm that smokers with MHP have similar motivation to quit smoking and are at the same stages of change as smokers without MHP, but have less confidence in their ability to quit, which seems to indicate reduced self-efficacy to quit (18, 19, 63). In contrast to Cruvinel et al. (64), who reported fewer quit attempts in people with MHP than in those without, our study showed a higher number of quit attempts in participants with MHP, which may be related to the lower self-efficacy mentioned above. Lubitz et al. (18) found an association between more quit attempts and lower nicotine dependence. In our study, people with MHP had both higher nicotine dependence and more quit attempts. This may be because higher nicotine dependence leads to more severe withdrawal symptoms, which increase the relapse risk, especially when combined with low self-efficacy in people with MHP (65). However, when combined with strong motivation to quit, this may also lead to more quit attempts.

Although differences in sociodemographic and smoking-related characteristics were observed between individuals with and without MHP, our regression analyses - adjusted for potential confounders - showed that experiencing MHP was no significant predictor of smoking cessation behavior. Significant associations were found with other variables (e.g., age, BMI and confidence in quitting), providing deeper insights into the determinants of cessation outcomes. Our regression analyses revealed that “confidence in quitting smoking” plays a crucial role in smoking behavior and quitting outcomes. Specifically, smokers with lower cigarette dependence tended to have more confidence in quitting, and higher confidence was associated with a greater likelihood of being ready to quit. Interestingly, those with higher self-confidence also reported more smoking-related health complaints, suggesting that health awareness may drive their determination to quit. These findings support the idea that smoking cessation programs should focus on building self-efficacy, especially among people with MHP, who often report lower confidence in quitting (19, 66). Interventions may also benefit from strategies to increase health awareness and address smoking-related health complaints to increase motivation to quit and improve cessation outcomes. Furthermore, our regression analysis showed an association between the older age of study participants and higher nicotine dependence, which is consistent with previous research (67). This may be due to a longer smoking history leading to increased nicotine tolerance and the gradual reinforcement of addictive behaviors over time (68). Long-term smokers often have ingrained smoking routines in their daily lives, making it more difficult for them to change their behavior. These findings underscore the need for targeted interventions for older smokers (67), focusing on increasing confidence in quitting and implementing age-specific quit strategies to improve cessation outcomes. Regression analysis also showed a negative association between BMI and readiness to quit smoking. This suggests that concerns about weight gain may influence the readiness to quit smoking. Previous research has noted that smokers often fear weight gain after quitting, which may reduce their motivation to quit (30, 69). As smoking is known to have appetite-suppressing effects (70, 71), individuals with a higher BMI may be more reluctant to quit smoking to maintain weight control strategy. To address this, smoking cessation programs should incorporate weight management strategies and promote healthy lifestyle habits to increase motivation and to support successful cessation (69). It is also important to recognize that readiness to quit smoking is linked to motivation, which can fluctuate over time (72–74). Longitudinal studies, including randomized controlled trials (RCTs), have shown that motivation to quit smoking is often variable, with individuals experiencing ambivalence and periods of decreased motivation (72, 74, 75). These fluctuations should be considered when designing interventions. Practical implications include offering flexible support mechanisms, such as booster sessions or follow-up interventions, and using motivational interviewing techniques to address ambivalence and reinforce commitment to quitting (74, 76). In addition, setting small, achievable goals and providing ongoing encouragement can help individuals overcome periods of low motivation and improve long-term success (77).

Our findings on the reasons for smoking are in line with previous research (37, 40, 44), but there were differences between the two groups. Smokers with MHP were significantly more likely to smoke for stress relief and mental health reasons, such as coping with negative emotions and managing symptoms of mental health disorders, supporting the self-medication hypothesis (9, 38, 44). However, recent evidence (78) indicates that smoking cessation is associated with reduced depression and anxiety and improved general mental health, highlighting the need to inform people with MHP about these benefits (44). In contrast, individuals without MHP were more likely to smoke for social and reward-related reasons, such as socializing or pleasure/stimulation. This aligns with research showing that socially integrated individuals are more influenced by their environment in their smoking behavior (79). Given the different but overlapping motives for smoking between the two groups, interventions for people with and without MHP should address social and habitual triggers, while people with MHP may benefit from an integrated mental health approach, as smoking is often a coping mechanism for psychological distress in this group (80). According to the transtheoretical model of behavior change (81), people who smoke for emotional regulation need targeted support to develop healthier coping mechanisms. Therefore, smoking cessation programs should integrate alternative coping strategies, like mindfulness or exercise, to facilitate behavior change (55). While behavioral support can be effective, cognitive-behavioral therapy techniques, including cognitive restructuring and reappraisal, may also be essential in addressing maladaptive emotion regulation (82).

The findings on barriers to smoking cessation in our study are also consistent with those of previous studies (42–44, 46). These barriers reflect the underlying and previously mentioned differences in reasons for smoking between individuals with and without MHP. Individuals with MHP, who smoked for addiction, behavioral-, stress- and mental health reasons, reported higher barriers for individual and lifestyle factors and cultural, socioeconomic and environmental issues (20). In contrast, people without MHP were more likely to report social and community network barriers (20), reflecting their smoking for social and reward reasons in addition to addiction and behavioral factors. These overlapping patterns suggest that the factors driving smoking behavior also contribute to the challenges of quitting. To effectively support smokers with MHP, it is also important to address external barriers and improve access to cessation programs by integrating them into existing structures such as mental health settings (20, 46). Integrated care models can improve accessibility and effectiveness by making smoking cessation part of routine mental health care. Training mental health professionals in smoking cessation techniques ensures that behavioral and pharmacological supports are seamlessly integrated into existing treatment plans (9). Additionally, social support theory (83) emphasizes the importance of peer and professional support in sustaining behavior change. Structured support groups, digital platforms, and personalized counseling can increase motivation, self-efficacy and reduce relapse risk (11, 84).

As in the studies by Sharapova et al. (41) and Sagayadevan et al. (45), the most common motives for quitting smoking in our study were health (intrinsic) and money (extrinsic factor). Other motives reported by both groups, such as physical fitness (42), family or partner (41, 42, 45) or pregnancy (45), can also be found in the literature. Importantly, no significant differences in the distribution of intrinsic or extrinsic motives were found between individuals with and without MHP. This is consistent with previous research indicating that both groups are similarly motivated to quit smoking (18, 63). In our study, health concerns were the main intrinsic motivator for quitting smoking among individuals with MHP. This probably reflects their heightened awareness of the negative physical and psychological effects of smoking (2), as they experienced more pronounced smoking-related complaints. Money was the second most common motive, possibly because the lower socioeconomic status and limited financial resources of people with MHP make the cost savings associated with quitting particularly attractive (37). However, intrinsic motives outweighed extrinsic motives in both groups, implicating a stronger role for intrinsic motives in the decision to quit smoking (85). The literature indicates that intrinsic motives are more likely to lead to long-term behavior change than extrinsic motives and that intrinsically motivated smokers are more likely to quit successfully (43, 85). Therefore, smoking cessation counselling should focus on strengthening intrinsic motives using motivational interviewing and personalized counseling (76). Additionally, extrinsic motivators, such as financial incentives, can serve as initial triggers for quit attempts (86).

Our study found similar patterns to those reported by Trainor and Leavey (46) in terms of smoking cessation support. While there were no significant differences in individual or social support between groups, people with MHP reported a greater need for structural support, including access to replacement products, cessation programs, and multiple cessation support options. The greater need for structural support among individuals with MHP can be understood in terms of the socio-ecological model (87, 88), which emphasizes the role of systemic and environmental factors in shaping behavior. Individuals with MHP may be more likely to rely on formal and professional support systems because these are consistent with the structured care they already receive for their mental health treatment, such as medication, therapy and counseling from health professionals (46). In contrast, people without MHP may have stronger informal support networks, making family support a more important factor in their quit attempts (79). However, lower social support among individuals with MHP suggests weaker informal networks or less encouragement from their environment (37, 89). As social and environmental support positively influences smoking cessation outcomes (90) and assisted quit attempts are more likely to be successful than unassisted attempts (91), it is important to ensure access to multiple forms of support for both groups. In particular, interventions should strengthen social connections for people with MHP, for example by integrating peer support groups into smoking cessation programs (92). Additionally, as only 4% of respondents without MHP and 12.8% of respondents with MHP in the present study received a medical recommendation to quit smoking, healthcare professionals should be trained to proactively offer smoking cessation counseling (9).

Looking at the preferences of our study participants for a smoking cessation program combined with exercise, we see that our respondents’ ideas partially match the characteristics of previously conducted studies. In the literature, studies can be found that were conducted over 8 (93), 9 (94), 10 (95) or 12 (96) weeks, a period that comes closest to the desired duration (10 or 11 weeks) of our respondents. Although there are previous studies including people with or without MHP that offered exercise units 2 or 3 days a week (29, 93, 96, 97), the smoking cessation programs were not offered 2–3 days a week, but mostly 1 day a week (94–96). While participants with MHP in our study preferred shorter exercise sessions of 48 min on average, those without MHP preferred 60-min exercise sessions. There are studies in the literature that have used 40-min exercise sessions as an adjunct to smoking cessation in people with MHP (93, 96). Trials have also been conducted in people without MHP using 60-min exercise units (27), but so far, more trials are using shorter exercise sessions (26). In the studies found in the literature, the majority of the exercise sessions were held indoors (24–26). In the present study, both groups of participants were able to imagine outdoor sessions in addition to indoor exercise. When looking at the types of sports preferred by the respondents, we see that people without MHP prefer more intensive forms of exercise, such as running, workouts, ball sports but also yoga. People with MHP tend to prefer less intensive exercise such as (Nordic) walking, but can also imagine dancing, workouts, swimming and yoga. Previous studies evaluating the effect of exercise combined with smoking cessation programs on smoking abstinence in people with MHP used aerobic activities such as walking (treadmill), running or cycling (24). In the trials that included people without MHP, the main type of exercise used was aerobic exercise, but isometric exercise, resistance exercise, and yoga were also used (26). Previous studies have also shown that moderate-intensity exercise led to greater reductions in cigarette cravings than low-intensity exercise (98). In addition, high exercise adherence was associated with higher smoking cessation rates in the meta-analysis by Zhou et al. (26). Since more than 70% of the surveyed participants with and without MHP expressed a general interest in participating in a smoking cessation program in combination with exercise, the preferences of the two groups in the present study should be considered when planning future programs to increase participation rates and adherence to the program. The feasibility of the preferences and the characteristics of previous studies that showed a positive effect should also be considered. However, engaging in exercise can be challenging for people with MHP, particularly those with severe mental illness (e.g., schizophrenia or bipolar disorder), due to negative symptoms and medication side effects such as sedation, weight gain and reduced heart rate variability (99, 100). Future programs should address these barriers by offering low and moderate intensity exercise sessions of shorter duration, based on participants’ preferences. Gradual progression, supervised sessions, personalized treatment recommendations, and flexible scheduling may further support adherence (99, 101). In addition, motivational strategies such as goal setting, self-monitoring and social support could improve long-term engagement in exercise (76, 77, 101). Furthermore, as people with MHP tend to have a higher BMI, as also reflected in the sociodemographic differences in this study, smoking cessation programs should address the specific challenges faced by this group (58). Physical and psychological barriers, such as limited mobility, discomfort or low self-efficacy, may make it more difficult to engage in exercise sessions (58). Offering low-impact exercise options, ensuring easy access, and creating a supportive and non-judgmental environment could enhance participation and long-term adherence (25, 32, 84).

The present study has several limitations that should be considered. First, the study used a cross-sectional design, which does not allow for causal inference. In addition, the sample was not representative of the general population, as recruitment was conducted through online platforms, self-help forums, and university mailing lists. This may have led to selection bias, as individuals without Internet access or those less involved in online communities were excluded. In addition, younger people were overrepresented in the sample, which further limits the generalizability of the findings. Future research should aim to recruit a more representative sample, possibly using stratified or randomized sampling methods to improve external validity. The distribution of participants was uneven, with 82 individuals reporting MHP compared to 175 without MHP. This imbalance may have influenced group comparisons, and future studies may benefit from a matched control strategy to improve comparability. To address potential confounding effects, multiple regression analyses were performed in this study. However, further research should include additional methods to control for confounding variables and strengthen the robustness of the findings. The study relied on self-reported data, which are subject to reporting bias and social desirability effects (102). Although the anonymous online survey format may have encouraged honest reporting, the accuracy of self-reported smoking behavior and mental health status remains a limitation. This self-report bias may have affected the validity of the results, particularly for sensitive topics such as mental health and smoking habits. In addition, due to privacy and ethical concerns, participants were asked a single question about whether they had a diagnosed mental health condition, rather than using standardized questionnaires. While this approach protected privacy, it may not have fully captured the range of mental health conditions, and the accuracy of self-reports may be limited. Furthermore, the nature and severity of symptoms of different mental health conditions may influence the type of support needed for smoking cessation and exercise participation. Future research should consider conducting secondary analyses to explore differences between specific diagnoses within the MHP group, allowing for more tailored interventions based on individual needs. Moreover, the lack of mandatory questions due to ethical guidelines resulted in variable sample sizes across analyses. The survey instruments, including self-report questionnaires, may not have comprehensively assessed complex psychosocial factors related to smoking cessation and mental health. More sophisticated instruments or qualitative methods in future studies could help to capture these nuances and reduce bias. A limitation regarding the categorization and clustering of “reasons to smoke,” “barriers,” “motivation,” and “support to quit” is that results may vary depending on how these categories are defined and classified. Although the categories were created based on established theory and literature, the choice of classification system may influence the results and should be considered when interpreting the results. Finally, while the study assessed participants’ preferred type of exercise related to smoking cessation, it did not assess their preferred intensity of exercise. Given that previous research has suggested that exercise intensity may influence smoking cessation success (98), future studies should include this variable to provide more nuanced insights into exercise-based smoking cessation programs.

## 5 Conclusion

The aim of the present study was to identify the reasons for smoking, barriers, motives, and support for smoking cessation, and to examine preferences for a smoking cessation program combined with exercise in smokers with self-reported mental health problems compared to smokers without reporting mental health problems. The results highlight the need for differentiated smoking cessation strategies tailored to individuals with MHP and emphasize the importance of addressing both mental and physical health challenges.

Based on the findings of our study, future smoking cessation programs for smokers with MHP should include personalized interventions that combine behavioral support, coping strategies, weight management strategies and targeted psychoeducation. Interventions should also focus on increasing self-efficacy and confidence, managing withdrawal symptoms, and incorporating behavioral strategies, such as physical activity, which may increase smoking cessation success (31, 103). Incorporating exercise into these programs, especially through short, frequent, and varied physical activities such as walking or light exercise, can not only increase participation and adherence but also serve as a cost-effective alternative to smoking that requires minimal equipment and effort and is easily accessible at any time (25, 32, 84). In addition to individual barriers, environmental and structural barriers also need to be addressed. Smoking cessation interventions should be implemented in mental health care settings where people with MHP are already receiving treatment, ensuring barrier-free access and the integration of evidence-based cessation methods (20, 46). Public health policies should support this integration by providing health professionals with the necessary training and tools to address the particular challenges that people with MHP face in quitting smoking (11, 46, 104). Comprehensive smoking cessation counseling by health professionals in mental health care settings that combines psychosocial support with smoking cessation strategies can more effectively promote long-term success (9). Further research, particularly clinical trials, will be essential to evaluate the effectiveness of tailored interventions and refine best practices for helping smokers with MHP quit smoking successfully. Furthermore, a mixed-method approach that combines both qualitative and quantitative data would provide valuable insights into the specific challenges faced by individuals with MHP and help improve the development and implementation of these programs.

## Data Availability

The datasets presented in this article are not readily available because of ethical guidelines. Requests to access the datasets should be directed to Stefanie Schöttl, Stefanie.Schoettl@uibk.ac.at.
