# Supplementary material for: Smoking cessation and exercise: perspectives from smokers with and without mental health problems
Source: Front Public Health. 2025 May 16;13:1589719. doi: 10.3389/fpubh.2025.1589719 (PMC12122771; doi:10.3389/fpubh.2025.1589719)
Supplement: Supplementary file 1 [file Table_1.docx]

Supplementary Material

The categories for reasons for smoking (Table S1), barriers (Table S2), motivation (Table S3) and support (Table S4) to quit smoking were defined based on theoretical frameworks, including behavioral theory (1), self-determination theory (2, 3), coping theory (4, 5), transtheoretical model of behavior change (6), as well as relevant literature.

The classification of smoking reasons was based on the aforementioned theoretical approaches and previous research (7–11). Based on these frameworks, reasons for smoking were grouped into five categories: 1) stress-/mental-related factors, 2) reward-related factors, 3) addiction-related factors, 4) social factors and 5) behavioral factors. The categorization of smoking cessation barriers in this study was based on the Smoking Cessation Barriers Model developed by Twyman et al. (12). This model, designed to identify barriers in vulnerable populations, is consistent with the social determinants of health framework and provides a structured approach to understanding barriers to smoking cessation. The four domains were used to categorize the reported barriers: 1) individual and lifestyle factors, 2) social and community networks, 3) living and working conditions and 4) cultural, socioeconomic and environmental factors. In addition, smoking cessation motives were categorized into intrinsic and extrinsic factors, following the approaches outlined by (13–15). Intrinsic motives reflect internal drivers and personal aspirations, whereas extrinsic motives stem from external drivers and rewards (13). To analyze the supportive factors for smoking cessation, three categories were established based on previous literature (12, 16–19): individual-level support, social support, and structural support.

**Supplementary Table 1:** Factors for each category of reasons for smoking among smokers without (group S) and with (group SMI) mental illness (multiple answers were possible)

| Reasons | Group S (n=175) | Group SMHP (n=82) | p-value |
| --- | --- | --- | --- |
| Stress-/mental-related factors |  |  |  |
| Stress | 54.3% | 61.0% | 0.313 |
| Coping with negative mental symptoms | 4.0% | 41.5% | **<0.001** |
| Reward-related factors |  |  |  |
| Pleasure | 68.6% | 45.1% | **<0.001** |
| Stimulation | 19.4% | 9.8% | 0.051 |
| Addiction-related factors |  |  |  |
| Addiction | 59.4% | 70.7% | 0.080 |
| Social factors |  |  |  |
| Sociability | 57.1% | 30.5% | **<0.001** |
| Smoking in a social environment | 30.3% | 13.4% | **0.004** |
| Behavioral factors |  |  |  |
| Habit | 72.0% | 65.9% | 0.316 |
| Boredom | 42.9% | 48.8% | 0.373 |
| Note: significant differences between groups measured by Chi²-test are shown in bold | | | |

**Supplementary Table 2:** Factors for each category of barriers to quit smoking among smokers without (group S) and with (group SMI) mental illness (multiple answers were possible)

| Barriers | Group S  (n=172) | Group SMHP  (n=79) | p-value |
| --- | --- | --- | --- |
| Individual and lifestyle factors |  |  |  |
| Habit | 70.9% | 55.7% | **0.018** |
| Strong craving | 51.2% | 60.8% | 0.156 |
| Fear of changes in personality and mood changes | 26.7% | 51.9% | **<0.001** |
| Fear of weight gain | 17.4% | 38.0% | **<0.001** |
| Sadness | 16.9% | 38.0% | **<0.001** |
| Low self-confidence | 13.4% | 35.4% | **<0.001** |
| Exhaustion | 5.8% | 22.8% | **<0.001** |
| social and community networks |  |  |  |
| Smoking in a social environment (family, friends) | 59.9% | 35.4% | **<0.001** |
| Parties | 51.2% | 20.3% | **<0.001** |
| Peer pressure | 14.5% | 7.6% | 0.121 |
| No support person | 2.3% | 8.9% | **0.019** |
| living and working conditions |  |  |  |
| Boredom | 34.3% | 35.4% | 0.860 |
| Stress | 56.4% | 57.0% | 0.933 |
| Lack of time | 2.3% | 0.0% | 0.172 |
| cultural, socioeconomic and environmental factors |  |  |  |
| Lack of appropriate local services | 2.3% | 11.4% | **0.003** |
| Lack of knowledge about smoking cessation programs | 3.5% | 10.1% | **0.033** |
| Note: significant differences between groups measured by Chi²-test are shown in bold | | | |

**Supplementary Table 3:** Factors for each category of motivation to quit smoking among smokers without (group S) and with (group SMI) mental illness (multiple answers were possible)

| Motivation | Group S (n=172) | Group SMHP (n=79) | p-value |
| --- | --- | --- | --- |
| Intrinsic factors |  |  |  |
| Health | 80.2% | 77.2% | 0.584 |
| Physical fitness | 41.3% | 29.1% | 0.064 |
| Extrinsic factors |  |  |  |
| Money | 40.7% | 46.8% | 0.361 |
| Family | 34.3% | 19.0% | **0.013** |
| Relationship/partner | 29.1% | 17.7% | 0.055 |
| Pregnancy | 2.9% | 2.5% | 0.867 |
| Note: significant differences between groups measured by Chi²-test are shown in bold | | | |

**Supplementary Table 4:** Factors for each category of support to quit smoking among smokers without (group S) and with (group SMI) mental illness (multiple answers were possible)

| Support | Group S  (n=172) | Group SMHP  (n=79) | p-value |
| --- | --- | --- | --- |
| Individual-level support |  |  |  |
| Distractions, other activities | 47.7% | 38.0% | 0.151 |
| Incentives (e.g. money) | 35.5% | 35.4% | 0.997 |
| Social Support |  |  |  |
| Family/friends | 36.6% | 20.3% | **0.009** |
| Physician | 9.9% | 31.6% | **<0.001** |
| Structural Support |  |  |  |
| Replacement products (e.g. chewing gum, e-cigarettes) | 17.4% | 29.1% | **0.035** |
| Access to local smoking cessation programs | 8.7% | 22.8% | **0.002** |
| Availability of various cessation support options | 8.1% | 26.6% | **<0.001** |
| Note: significant differences between groups measured by Chi²-test are shown in bold | | | |

REFERENCES

1. Rhodes RE, Boudreau P, Josefsson KW, Ivarsson A. Mediators of physical activity behaviour change interventions among adults: a systematic review and meta-analysis. *Health Psychol Rev* (2021) **15**:272–86. doi:10.1080/17437199.2019.1706614

2. Darabseh MZ, Selfe J, Morse CI, Aburub A, Degens H. Does Aerobic Exercise Facilitate Vaping and Smoking Cessation: A Systematic Review of Randomized Controlled Trials with Meta-Analysis. *Int J Environ Res Public Health* (2022) **19**:14034. doi:10.3390/ijerph192114034

3. Ussher MH, Faulkner GE, Angus K, Hartmann-Boyce J, Taylor AH. Exercise interventions for smoking cessation. *Cochrane Database Syst Rev* (2019) **2019**. doi:10.1002/14651858.CD002295.pub6.

4. Lazarus RS, Folkman S. Transactional theory and research on emotions and coping. *European Journal of Personality* (1987) **1**:141–69. doi:10.1002/per.2410010304

5. Treviño LA, Baker L, McIntosh S, Mustian K, Seplaki CL, Guido JJ, et al. Physical activity as a coping strategy for smoking cessation in mid-life and older adults. *Addict Behav* (2014) **39**:885–8. doi:10.1016/j.addbeh.2014.01.014

6. Prochaska JO, DiClemente CC. Stages and processes of self-change of smoking: toward an integrative model of change. *J Consult Clin Psychol* (1983) **51**:390–5. doi:10.1037/0022-006X.51.3.390

7. Jones CM, Schüz B. Stable and momentary psychosocial correlates of everyday smoking: An application of Temporal Self-Regulation Theory. *J Behav Med* (2022) **45**:50–61. doi:10.1007/s10865-021-00248-4

8. Ponciano-Rodríguez G, Valerio-Gutiérrez R, Pliego-Rosas C, Córdova-Alcaráz A. Development and Evaluation of a New Self-Rating Test to Assess the Psychological Dependence on Smoking (TAPDS). *Journal of Addiction Medicine and Therapy* (2015) **3**:1016.

9. Rocha SA, Hoepers AT, Fröde TS, Steidle LJ, Pizzichini E, Pizzichini MM. Prevalence of smoking and reasons for continuing to smoke: a population-based study. *J. bras. pneumol.* (2019) **45**:e20170080. doi:10.1590/1806-3713/e20170080

10. Russell MA, Peto J, Patel UA. The Classification of Smoking by Factorial Structure of Motives. *Journal of the Royal Statistical Society. Series A (General)* (1974) **137**:313. doi:10.2307/2344953

11. Tomkins SS. Psychological model for smoking behavior. *Am J Public Health Nations Health* (1966) **56**:Suppl 56:17-20. doi:10.2105/ajph.56.12_suppl.17

12. Twyman L, Bonevski B, Paul C, Bryant J. Perceived barriers to smoking cessation in selected vulnerable groups: a systematic review of the qualitative and quantitative literature. *BMJ Open* (2014) **4**:e006414. doi:10.1136/bmjopen-2014-006414

13. Curry S, Wagner EH, Grothaus LC. Intrinsic and extrinsic motivation for smoking cessation. *J Consult Clin Psychol* (1990) **58**:310–6. doi:10.1037/0022-006X.58.3.310

14. Curry SJ, McBride C, Grothaus L, Lando H, Pirie P. Motivation for smoking cessation among pregnant women. *Psychol Addict Behav* (2001) **15**:126–32. doi:10.1037//0893-164x.15.2.126

15. Gill KK, van der Moolen S, Bilal S. Phenomenological insight into the motivation to quit smoking. *J Subst Abuse Treat* (2021) **131**:108583. doi:10.1016/j.jsat.2021.108583

16. Das S, Prochaska JJ. Innovative approaches to support smoking cessation for individuals with mental illness and co-occurring substance use disorders. *Expert Rev Respir Med* (2017) **11**:841–50. doi:10.1080/17476348.2017.1361823.

17. Gu M, Li X, Qin T, Qiao K, Bai X, Wang Y, et al. Environment and social support for smoking cessation among community smokers in Beijing, China. *Tob Induc Dis* (2023) **21**:145. doi:10.18332/tid/172216

18. Trainor K, Leavey G. Barriers and Facilitators to Smoking Cessation Among People With Severe Mental Illness: A Critical Appraisal of Qualitative Studies. *NICTOB* (2017) **19**:14–23. doi:10.1093/ntr/ntw183

19. Visser JE, Rozema AD, Kunst AE, Kuipers MA. Smoking Cessation Support in Social and Community Service Organizations: Potential Activities, Barriers, and Facilitators. *NICTOB* (2024) **26**:922–30. doi:10.1093/ntr/ntae004
